# Membranous and Diphtheritic Conjunctivitis

**Published:** 1884-08

**Authors:** A. A. Hubbell

**Affiliations:** Professor of Diseases of the Eye, Ear and Throat, in the Medical Department of Niagara University


### Membranous and Diphtheritic Conjunctivitis.

By A. A. Hubbell, M. D.,

Professor of Diseases of the Eye, Ear and Throat, in the Medical Department of Niagara University.

*Case* 1. Mr. C. H., aged 22, applied for treatment at the Good Samaritan Eye and Ear Infirmary in April, 1883, with the complaint that "something was in his right eye." His general appearance was anæmic, and he was debilitated and suffering from a chronic cough, the nature of which I did not investigate. His left eye was healthy. The right eye, which, he said, had "something in it that had been there three or four days," presented the external appearance of a simple conjunctivitis of a moderately severe type. There was muco-purulent discharge, with lachrymation and photophobia. The eyelids were somewhat swelled, and the ocular conjunctiva was reddened. On eversion, the palpebral conjunctiva of the lower lid was red and "papillated," and was secreting muco-pus. That of the upper lid was also red, and the papillæ at the retro-tarsal were enlarged, and over the portion covering the tarsal cartilage was a thin, pellucid, membraniform substance. While everting the lid this became slightly loosened and corrugated near the external canthus, and I took a forceps and gently removed it entire, leaving the conjunctiva with a more reddened appearance and bleeding slightly at two or three points. The pseudo-membrane was almost transparent, thin, and in size was about that of the tarsal cartilage, extending from the outer to the inner extremities and from the margin of the lid to near the upper retro-tarsal fold.

The eye was dressed with a compress moistened with a solution of boracic acid of the strength of ten grains to an ounce of water, an instillation of the same having also been made in the eye. The instillations were to be repeated every two hours, and the compress was to be kept constantly applied and wet with the solution.

The patient returned on the next day with all the symptoms abated. The pseudo-membrane had re-formed, however, but only over about one-half the inner surface of the tarsal portion of the upper lid toward the inner canthus. I again removed it with the forceps, easily, leaving one bleeding point, and the conjunctiva beneath reddened as before. The exudate was not as thick as before, and was less perfect in formation, although distinctly membranous. The treatment previously pursued was continued.

On the following day, the third, of treatment, the membranous formation could not be seen, and all the symptoms were much improved. The compresses, wet with the solution, were continued, and the instillations of the same were directed to be made every four hours.

In eight days after beginning treatment the patient was discharged, with the eye cured.

*Case* 2. J. W., aged 31, clerk in a grocery, presented himself at my office June 9, 1884, with an affection of his right eye, which began two days before. On examination, I found the eye much reddened from deep scleral injection, as well as from some injection of the vessels of the ocular conjunctiva. The lids were normal in appearance, with the exception of some redness of their inner surfaces. There was no muco-purulent discharge, but there was much lachrymation, and photophobia was intense. On the cornea, near the centre of its anterior surface, was an ulcer, circular in shape, somewhat excavated, and about two millimetres in diameter. The pupil was contracted, and but slightly responsive to light. The iris, however, was not discolored or otherwise abnormal in appearance. The eyeball was tender and the patient suffered some ciliary pain. The general appearance of the patient was healthy, and no disease could be discovered, besides the corneal ulcer of the right eye, except a slight gonorrhœa, which had been in progress a few days. The left eye was healthy.

I directed the eye to be carefully bandaged, and gave a solution of atropia, two grains to an ounce of water, to be instilled into the eye every four hours.

June 11th. The patient returned. The subjective symptoms were about the same as at the previous visit, but the objective symptoms were much changed and greatly out of proportion to the subjective ones. The discharge, which previously was lachrymal alone, was now muco-purulent, also, but only in a moderate degree. The lids were red and swelled, but not painful. The eyeball was of a deeper red color than before, and the ocular conjunctiva was considerably raised around the margin of the cornea. The ulcer at the central part of the cornea had enlarged in diameter a little, and added to this was another ulcer, at the lower edge of the cornea, about one millimetre in width, and extending along its margin for a distance of nearly one centimetre. The cornea was also considerably hazy, especially in its lower half. The iris had not responded to the action of the atropia, the pupil being yet contracted. On eversion of the upper lid, its inner surface was intensely red, and the papillæ were much enlarged, and the conjunctiva, particularly of the upper portion of the eyeball, was chemotic. The inner surface of the lower lid and the contiguous conjunctival covering of the eyeball were thickened, the thickening increasing toward the lower retro-tarsal fold, where it was greatest. The appearance was precisely like moistened chamois leather, yellowish-gray in color, and apparently non-secreting, with the grooved markings on the inner surface of the lower lid, usually seen when the conjunctival papillæ are enlarged, quite indistinct. In other words, the conjunctiva of the eyeball below the lower margin of the cornea and that of the lower lid and corresponding retro-tarsal fold looked as if it had been deeply seared with hot metal. I was greatly surprised to find the eye in this condition, and my prognosis was not very cheering.

As to treatment, I determined to use antiseptic remedies. Among those which can be applied to the eye, I regard boracic acid as the best, by far. I therefore prescribed this for my patient, of the strength of ten grains to an ounce of water. I ordered a few drops to be instilled into the lower conjunctival sac every half hour, and to keep linen, folded two or three times, thoroughly wet with the solution and constantly applied over the lids. Strict cleanliness was also enjoined.

June 12th, 8.00 A. M. The eye is about the same. There is very little pain, and the muco-purulent discharge is a little more in quantity. The lower lid, on its inner surface, and the lower part of the eyeball, present the same thickened, soggy, ash-gray or yellowish-gray appearance. The condition of the cornea and its ulcers is unchanged. The boracic acid treatment is to be continued.

Patient was again seen at 7.00 P. M. The conjunctiva is less thickened and discolored near the edge of the lower lid and near the lower margin of the cornea. Other symptoms are about the same. The treatment is not changed. A slight conjunctivitis has begun in the left eye, for which I prescribe the same solution.

June 13th, 8.00 A. M. All the symptoms are better. The lids are less swelled, the corneal ulcers are healing, photophobia and lachrymation are diminished, no pain. The exudation is disappearing by a sort of breaking down or softening process, with an increased secretion of pus. The parts now covered by it are at the retro-tarsal fold and toward the inner canthus. Where it has disappeared, the conjunctiva seems almost destroyed. The left eye is better. No change is made in the treatment. Same day, at 7.00 P. M., the patient is still better in every respect.

June 14th. Patient was seen twice. The chemosis has subsided, the corneal ulcers are nearly healed, the lids are not swelled, and the exudation has entirely disappeared, apparently by softening and suppuration. At the lower retro-tarsal fold the conjunctiva is entirely destroyed, the mucous surfaces are granulating, and adhesions are taking place between the opposing surfaces of the lower lid and eyeball. This granulating process is confined to and near the retro-tarsal fold. There is considerable discharge of pus from the eye. The boracic acid solution is continued, but not instilled as frequently.

From this date the eye rapidly recovered. At the end of the next twenty-four hours the corneal ulcers were healed, the discharge was much diminished, and the lower conjunctiva was appearing more normal, except where entirely destroyed near and at the retro-tarsal fold. The patient was discharged June 19th, ten days after beginning treatment, with all diseased conditions removed, excepting obliteration of the lower conjunctival sac or fold, and a corresponding adhesion of the lid to the eyeball. The adhesion, however, was not sufficient to materially obstruct the movements of the eye and lids, the inner surface of the lower lid being free for one-fourth to one-third of an inch from the margin.

*Remarks.*—I present these two cases to my readers because of the infrequency of these forms of conjunctival inflammation, and because of the apparently remarkable effects of the boracic acid treatment.

The membranous formation, illustrated by the first case, may supervene upon an ordinary, or more generally, a purulent conjunctivitis, the attending secretion becoming to a degree plastic, and assuming an appearance varying from mere shreds or patches to a complete covering of the mucous membrane. It appears to be incidental in conjunctivitis to certain ill-defined, local and general conditions of the patient, being seen more frequently in strumous and anæmic children and adults.

The truly diphtheritic conjunctivitis, as shown in the second case, is more than a mere filmy, flaky or shreddy exudation upon the surface of the mucous membrane. It is a thorough, and often deep, infiltration of plastic matter into the conjunctival and contiguous tissues, and involves their integrity in proportion to its amount and extent. It is not, however, a diphtheria affecting the eye in the specific sense in which diphtheria is usually understood; for, excepting when arising as a mere extension of the diphtheritic process in the throat or nose, it has nothing essentially specific, either in its general character or ætiology. It is simply an exudative inflammation of the conjunctiva, having the local, but not the general, expression of diphtheria, whence its name.

It is proper, furthermore, to distinguish membranous from diphtheritic conjunctivitis, as their characteristics, symptoms and complications are widely different, as seen by the above cases. The one is more like a collected secretion, while the other is an infiltration. The one is confined to the surface, and is not usually dangerous, while the other extends more or less deeply, compresses the blood vessels and lymphatics, and by the arrest of the supply of nutrition to the cornea and other structures, endangers their vitality.

The causes of the exudative forms of conjunctivitis are those of the catarrhal or purulent varieties. In the first case, above noted, the membranous formation was incidental, apparently, to an ordinary conjunctivitis; in the second, or the diphtheritic case, the gonorrhœal virus, it would seem, may have been a causative element. Whatever the cause of these forms of inflammation may be, it usually acts upon both eyes. In each of my cases, however, only one eye was effected, although in the second case the second eye began to become inflamed, but the disease was soon aborted, as I believe, by the boracic acid treatment applied to it.

The course and results of diphtheritic conjunctivitis are often very destructive, particularly to the cornea, and at one time in my case I feared that this structure would certainly become necrosed and end in perforation and loss of sight. The pain, which is genrally very severe, was not as great as would be expected with the two corneal ulcers, the amount of swelling of the lids and the chemosis which existed.

The treatment in this class of cases, which usually consists of cold or warm applications, quinine and carbolic acid lotions, astringents, atropine, etc., does not always yield the most happy results. Boracic acid has been recommended, but it seems to me in too weak solutions.

For several years I have used this drug as an antiseptic in the treatment of diseases and wounds of the eye and ear with a great deal of satisfaction; but I find it of much greater benefit when employed in substance in impalpable powder, or at least in a saturated solution. As an antiseptic to the eye, especially, it has the advantage of not being irritating to its mucous membranes like carbolic acid, bichloride of mercury, etc. In cataract and other operations upon the eye, I constantly employ it, and, as I believe, advantageously.

In using it in the above cases, in the second particularly, its efficacy was most apparent. In both cases the disease was promptly arrested and an excellent recovery took place. In the diphtheritic case, which seemed to be growing worse rapidly, the disease was stayed at once on beginning its application, the infiltration and exudation ceased, the swelling of the lids and conjunctiva began to lessen within twenty-four hours, the corneal ulcers and haziness did not extend, but soon began to improve, the pain was less severe, and, indeed, the rapid recovery which followed was remarkable. Furthermore, the same treatment arrested a commencing conjunctivitis in the second case.

